# Supplementary material for: Comparison of the Effects of DOTA and NOTA Chelators on 64Cu-Cudotadipep and 64Cu-Cunotadipep for Prostate Cancer
Source: Diagnostics (Basel). 2023 Aug 11;13(16):2649. doi: 10.3390/diagnostics13162649 (PMC10453766; doi:10.3390/diagnostics13162649)

Supplementary Materials

Comparison of the effects of DOTA and NOTA chelators on ^{64}Cu-cudotadipep and ^{64}Cu-cunotadipep for prostate cancer

Inki Lee[1,†], Min Hwan Kim[2,†], Kyongkyu Lee[2], Keumrok Oh[2], Hyunwoo Lim[2], Jae Hun Ahn[3,4], Yong Jin Lee[3], Gi Jeong Cheon[5], Dae Yoon Chi[2,*], Sang Moo Lim[1,*]

[1] Department of Nuclear Medicine, Korea Institute of Radiological & Medical Sciences, Seoul 01812, Republic of Korea

[2] Research Institute of Radiopharmaceuticals, FutureChem Co., Ltd., Seoul 04793, Republic of Korea

[3] Division of Applied RI, Korea Institute of Radiological & Medical Sciences, Seoul 01812, Republic of Korea

[4] Graduate School of Translational Medicine, Seoul National University College of Medicine, Seoul 03080, Republic of Korea

[5] Department of Nuclear Medicine, Seoul National University College of Medicine, Seoul 03080, Republic of Korea

[*]Correspondence: slim0328@nate.com, daeyoon.chi@futurechem.co.kr; Tel.: (82-2-970-2241 (S.M.L), 82-2-497-3114(D.Y.C))

[†]These authors contributed equally.

Table S1. *In vitro* stability of ^{64}Cu-cudotadipep and ^{64}Cu-cunotadipep.

	^{64}Cu-cudotadipep		^{64}Cu-cunotadipep	
Time (hour)	Human serum	Mouse serum	Human serum	Mouse serum
2	99.12±1.02	99.50±0.09	97.98±0.23	98.28±0.39
24	99.63±0.14	99.67±0.43	99.11±0.22	99.61±0.16
48	99.84±0.28	99.49±0.08	98.43±0.18	98.27±0.51

Table S2. Dosimetry of ^{64}Cu-cudotadipep and ^{64}Cu-cunotadipep based on biodistribution results in normal BALB/c mice.

	Absorbed dose (mGy/MBq)	
	^{64}Cu-cudotadipep	^{64}Cu-cunotadipep
Brain	$5.03 \times 10^{-2} \pm 6.34 \times 10^{-3}$	$2.46 \times 10^{-3} \pm 3.46 \times 10^{-4}$
Intestine	$3.92 \times 10^{-1} \pm 1.02 \times 10^{-1}$	$3.25 \times 10^{-2} \pm 2.23 \times 10^{-3}$
Stomach	$4.71 \times 10^{-1} \pm 2.54 \times 10^{-1}$	$3.69 \times 10^{-2} \pm 3.57 \times 10^{-3}$
Heart	$1.32 \pm 4.96 \times 10^{-1}$	$9.12 \times 10^{-2} \pm 7.58 \times 10^{-3}$
Kidneys	8.10 ± 1.49	$2.02 \pm 2.44 \times 10^{-1}$
Liver	$1.27 \pm 4.32 \times 10^{-1}$	$4.96 \times 10^{-2} \pm 3.89 \times 10^{-3}$
Lungs	$7.67 \times 10^{-1} \pm 2.93 \times 10^{-1}$	$5.69 \times 10^{-2} \pm 1.01 \times 10^{-2}$
Muscle	$4.05 \times 10^{-2} \pm 9.16 \times 10^{-3}$	$5.62 \times 10^{-3} \pm 5.56 \times 10^{-4}$
Spleen	$1.54 \pm 2.83 \times 10^{-1}$	$3.27 \times 10^{-1} \pm 1.68 \times 10^{-2}$
Effective dose (mSv/MBq)	$3.67 \times 10^{-1} \pm 1.39 \times 10^{-1}$	$3.00 \times 10^{-2} \pm 2.16 \times 10^{-3}$

Table S3. Tumor to non-target ratio of ^{64}Cu-cudotadipep (A) and ^{64}Cu-cunotadipep (B), analyzed by biodistribution data

A. ^{64}Cu-cudotadipep

	2 h	6 h	24 h	48 h
Tumor to blood	2.29±0.39	3.06±1.27	7.20±1.59	6.88±1.14
Tumor to muscle	10.23±1.57	14.94±7.32	20.50±8.17	9.39±3.22
Tumor to liver	0.28±0.03	0.26±0.12	0.62±0.10	0.65±0.05
Tumor to kidney	0.35±0.06	0.27±0.13	0.59±0.11	0.43±0.05

B. ^{64}Cu-cunotadipep

	2 h	6 h	24 h	48 h
Tumor to Blood	2.37±1.50	8.75±0.78	32.17±10.36	27.20±12.28
Tumor to Muscle	11.48±9.31	25.12±3.13	90.90±17.61	38.33±20.42
Tumor to Liver	2.76±1.79	4.99±0.64	5.30±0.23	4.87±1.09
Tumor to Kidney	0.37±0.25	0.38±0.07	0.74±0.07	0.56±0.24

Figure S1. Comparison of PSMA binding affinity of ^{64}Cu-cudotadipep and ^{64}Cu-cunotadipep in PSMA-positive 22Rv1 cells.

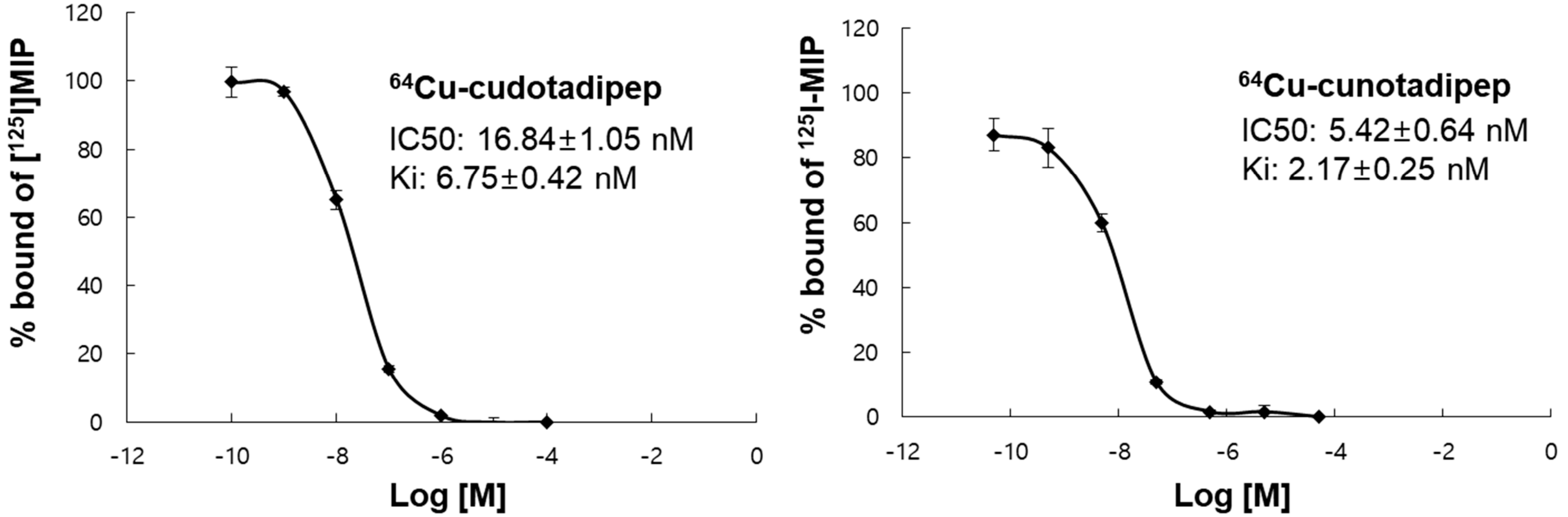

Figure S2. Comparison of uptakes of ^{64}Cu-cudotadipep and ^{64}Cu-cunotadipep in PSMA-positive prostate cancer cells (PC3-PIP) and PSMA-negative prostate cancer cells (PC3-flu).

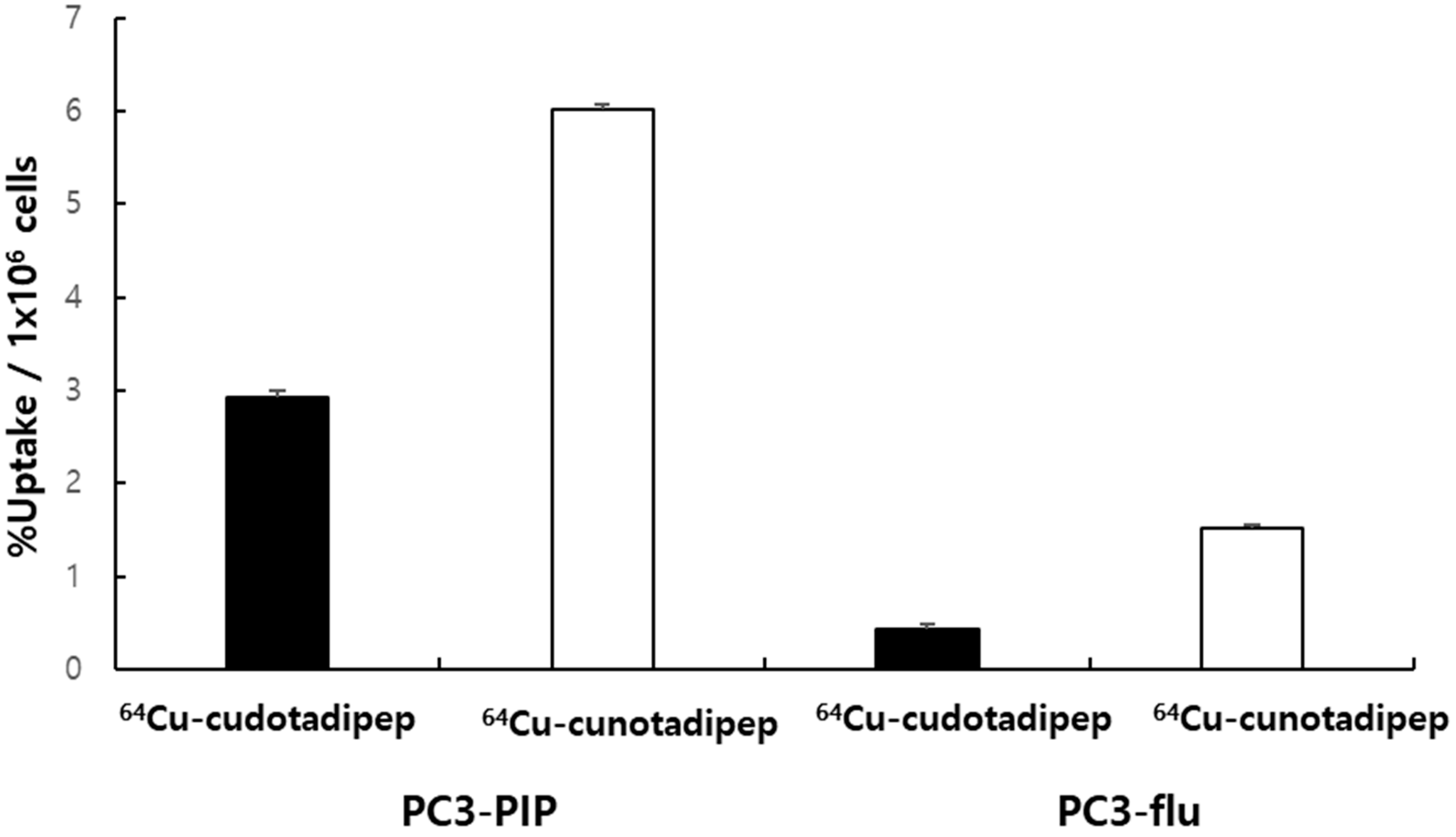

Figure S3. Radio-TLC results of *in vitro* stability in human and mouse serum of ^{64}Cu-cudiotadipep and ^{64}Cu-cunotadipep

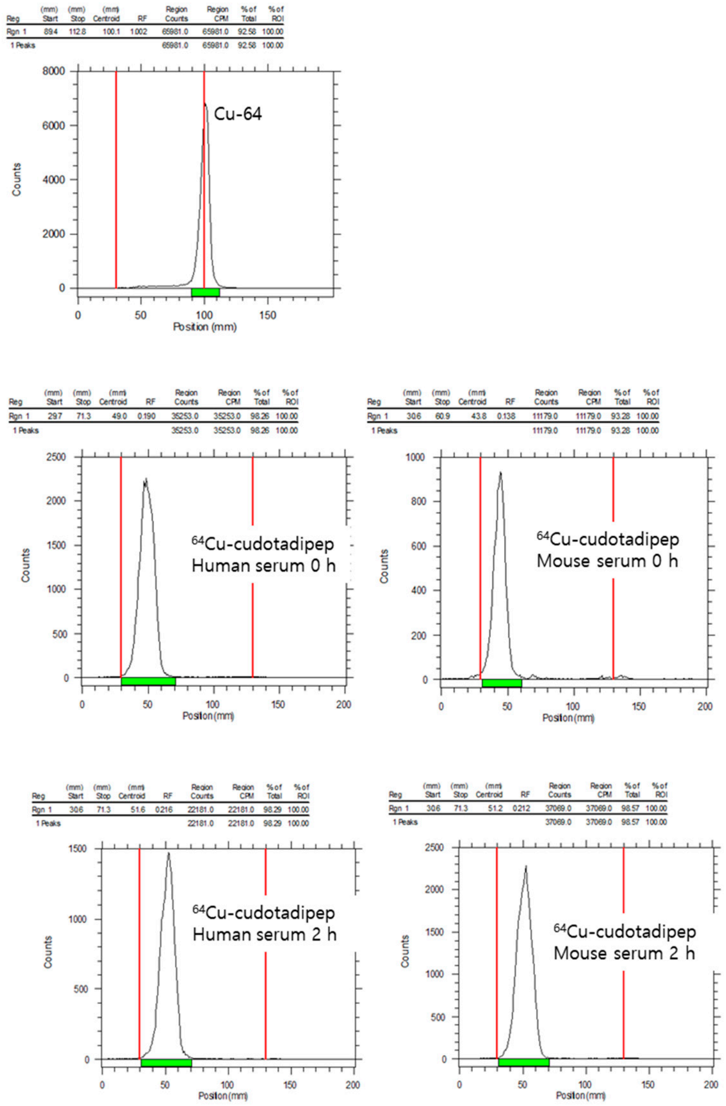

Reg	(mm) Start	(mm) Stop	(mm) Centroid	RF	Region Counts	Region CPM	% of Total	% of ROI
Rgn 1	30.6	75.6	54.8	0.248	23919.0	23919.0	97.95	100.00
1 Peaks					23919.0	23919.0	97.95	100.00

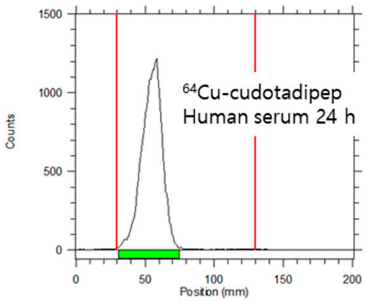

Reg	(mm) Start	(mm) Stop	(mm) Centroid	RF	Region Counts	Region CPM	% of Total	% of ROI
Rgn 1	29.7	60.9	44.2	0.142	5450.0	5450.0	94.45	100.00
1 Peaks					5450.0	5450.0	94.45	100.00

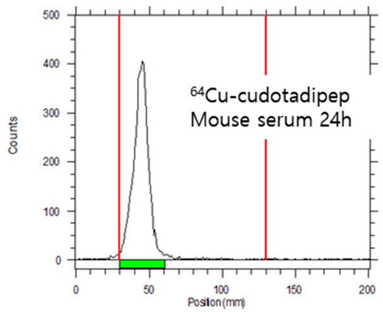

Reg	(mm) Start	(mm) Stop	(mm) Centroid	RF	Region Counts	Region CPM	% of Total	% of ROI
Rgn 1	30.6	66.9	45.6	0.156	3449.0	3449.0	96.75	100.00
1 Peaks					3449.0	3449.0	96.75	100.00

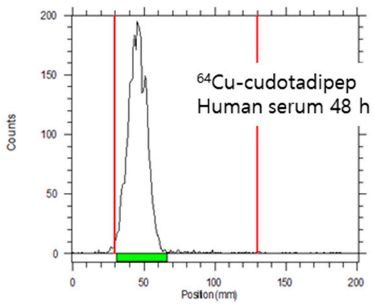

Reg	(mm) Start	(mm) Stop	(mm) Centroid	RF	Region Counts	Region CPM	% of Total	% of ROI
Rgn 1	30.6	73.9	52.4	0.224	8626.0	8626.0	96.91	100.00
1 Peaks					8626.0	8626.0	96.91	100.00

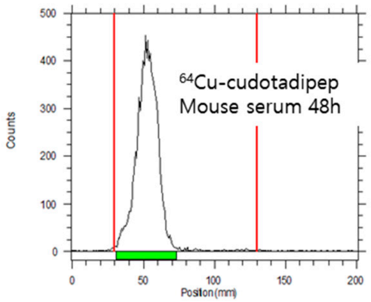

Reg	(mm) Start	(mm) Stop	(mm) Centroid	RF	Region Counts	Region CPM	% of Total	% of ROI
Rgn 1	89.4	112.8	100.1	1.002	65981.0	65981.0	92.58	100.00
1 Peaks					65981.0	65981.0	92.58	100.00

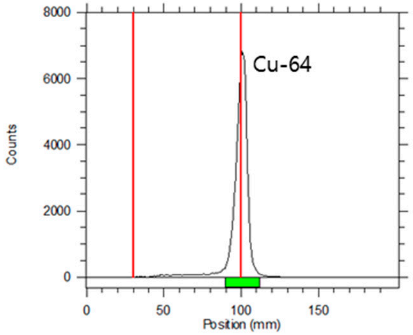

Reg	(mm) Start	(mm) Stop	(mm) Centroid	RF	Region Counts	Region CPM	% of Total	% of ROI
Rgn 1	29.7	63.5	42.6	0.126	20077.0	20077.0	91.29	100.00
1 Peaks					20077.0	20077.0	91.29	100.00

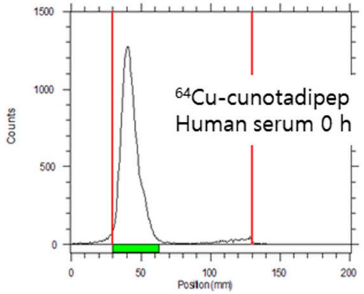

Reg	(mm) Start	(mm) Stop	(mm) Centroid	RF	Region Counts	Region CPM	% of Total	% of ROI
Rgn 1	30.6	66.1	42.2	0.122	39942.0	39942.0	96.13	100.00
1 Peaks					39942.0	39942.0	96.13	100.00

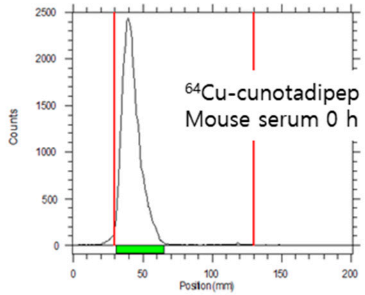

Reg	(mm) Start	(mm) Stop	(mm) Centroid	RF	Region Counts	Region CPM	% of Total	% of ROI
Rgn 1	30.6	68.7	44.4	0.144	57762.0	57762.0	95.14	98.03
Rgn 2	114.5	142.2	127.7	0.977	1161.0	1161.0	1.91	1.97
2 Peaks					58923.0	58923.0	97.05	100.00

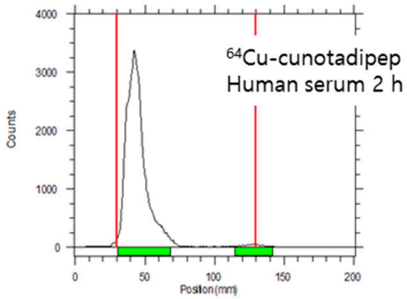

Reg	(mm) Start	(mm) Stop	(mm) Centroid	RF	Region Counts	Region CPM	% of Total	% of ROI
Rgn 1	29.7	60.9	41.1	0.101	65502.0	65502.0	96.57	98.57
Rgn 2	124.9	145.7	135.4	0.958	949.0	949.0	1.40	1.43
2 Peaks					66451.0	66451.0	97.97	100.00

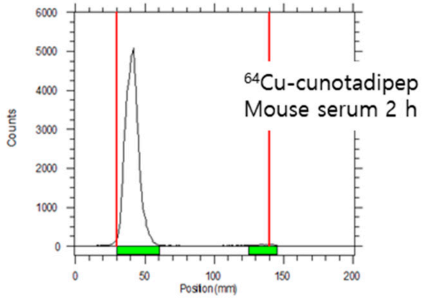

Reg	(mm) Start	(mm) Stop	(mm) Centroid	RF	Region Counts	Region CPM	% of Total	% of ROI
Rgn 1	29.7	71.3	45.2	0.152	61747.0	61747.0	96.75	100.00
1 Peaks					61747.0	61747.0	96.75	100.00

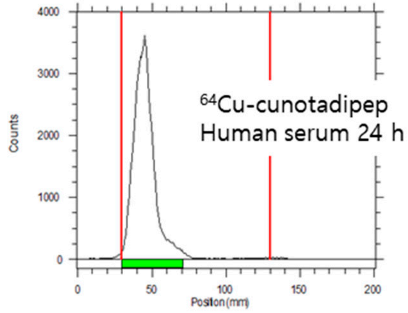

Reg	(mm) Start	(mm) Stop	(mm) Centroid	RF	Region Counts	Region CPM	% of Total	% of ROI
Rgn 1	29.7	71.3	48.0	0.180	17953.0	17953.0	95.90	100.00
1 Peaks					17953.0	17953.0	95.90	100.00

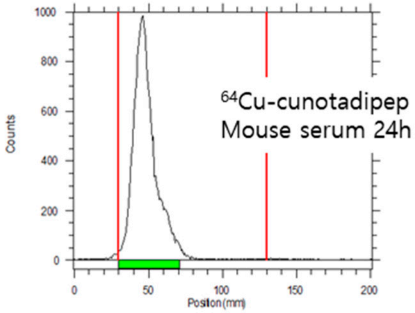

Reg	(mm) Start	(mm) Stop	(mm) Centroid	RF	Region Counts	Region CPM	% of Total	% of ROI
Rgn 1	29.7	61.7	44.1	0.141	4870.0	4870.0	94.09	100.00
1 Peaks					4870.0	4870.0	94.09	100.00

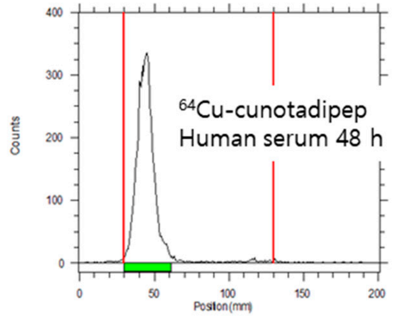

Reg	(mm) Start	(mm) Stop	(mm) Centroid	RF	Region Counts	Region CPM	% of Total	% of ROI
Rgn 1	26.3	57.4	38.7	0.087	4767.0	4767.0	94.58	100.00
1 Peaks					4767.0	4767.0	94.58	100.00

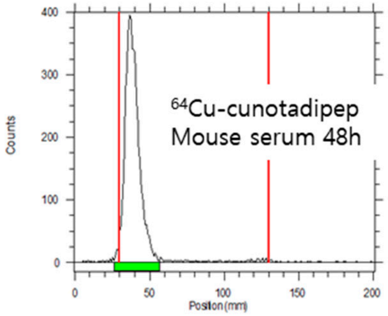

Figure S4. Comparison of ^{64}Cu-cudotadipep (A) and ^{64}Cu-cunotadipep (B) in major organs over time using a xenograft athymic nude BALB/c mice with PC3-PIP

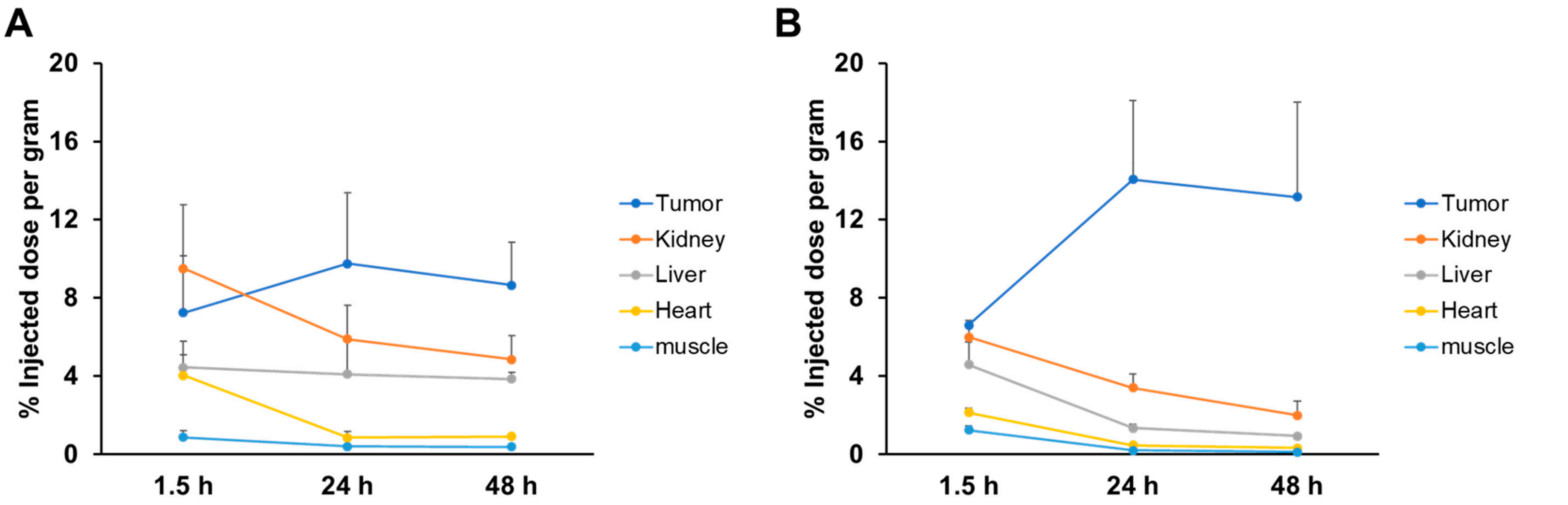

Supplement: Supplementary file 1 [file diagnostics-13-02649-s001.zip › diagnostics-2517033-supplementary.pdf]
